# Adaptation of the internal political efficacy scale and its associations with empathy, mental well-being, and global citizenship: a parallel mediation model

**DOI:** 10.3389/fpsyg.2026.1967651

**Published:** 2026-10-14

**Authors:** Mustafa Demirci

**Affiliations:** Department of Management and Organization, Yusufeli Vocational School, Artvin Coruh University, Artvin, Türkiye

**Keywords:** empathy, global citizenship, internal political efficacy, mental well-being, parallel mediation, structural equation modelling

## Abstract

Internal political efficacy, a significant psychological variable related to individuals’ perception of their ability to understand political processes and act effectively within them, has been gaining importance in the context of global citizenship. This study primarily examined whether empathy and mental well-being constitute parallel indirect pathways between internal political efficacy and global citizenship and, secondarily, evaluated the psychometric properties of the short form of the Internal Political Efficacy Scale in a Turkish sample. The research used two independent samples comprising a total of 681 participants: 311 participants for the psychometric analyses of the adapted scale and 370 participants for testing the structural model. In the psychometric analyses, confirmatory factor analysis verified the proposed structure of the scale and demonstrated that the instrument possesses acceptable reliability. In the structural model analyses, internal political efficacy was positively associated with empathy, mental well-being, and global citizenship. Moreover, empathy and mental well-being showed statistically supported parallel indirect pathways in the relationship between internal political efficacy and global citizenship, while the direct association remained significant. The study contributes to the global citizenship literature by integrating political efficacy, socio-emotional functioning, and mental well-being within a single psychological framework.

## 1 Introduction

The visibility of environmental, social, economic, and political problems that transcend national borders limits the consideration of citizenship solely within the framework of national belonging and obligations. Global citizenship education and global competence approaches emphasize individuals’ critical evaluation of local, national, and global issues, engagement with diverse cultural perspectives, and assumption of responsibility for a more just, peaceful, and sustainable world (Organisation for Economic Co-operation and Development [OECD], 2018; United Nations Educational, Scientific and Cultural Organization [UNESCO], 2015). Nevertheless, mere awareness of global problems or considering them as significant does not, in itself, entail the assumption of public responsibility. The ability of individuals to transform their knowledge and values into a citizenship orientation may also be related to perceiving themselves as competent to understand political and public issues and to contribute to these processes. Within this context, internal political efficacy refers to the subjective perception of competence in understanding political issues and participating effectively in political life (Niemi et al., 1991). Internal political efficacy has been shown to function as an important psychological mechanism in transforming political knowledge into participatory behaviors (Reichert, 2016). In the context of global citizenship, political self-efficacy has also been reported to be associated with global citizenship outcomes (Liu et al., 2023).

This study proposes to explain the relationship between internal political efficacy and global citizenship through two psychological processes, namely empathy and mental well-being. Empathy is a capacity comprising cognitive and emotional components that enable sensitivity to others’ emotions, perspectives, and needs (Davis, 1983). Recent research in the field of global citizenship suggests that cognitive empathy and global citizenship identity serve to foster individuals’ interest, engagement, and sense of responsibility toward global issues (Carmona et al., 2024). Mental well-being, as a form of positive psychological functioning, may represent a distinct resource relevant to sustained engagement with public and global issues. Findings from studies on civic engagement and well-being, which highlight the importance of meaning in life, self-efficacy, political efficacy, and contextual factors, also support this assumption (Chan et al., 2021; Fenn et al., 2021).

Although the existing literature demonstrates various dyadic relationships among these variables, studies that simultaneously test internal political efficacy, empathy, mental well-being, and global citizenship within a single structural model remain limited. Moreover, there is a need for psychometric evidence regarding the Turkish form of the four-item Internal Political Efficacy Scale developed by Niemi et al. (1991). This research therefore primarily examines the direct relationship between internal political efficacy and global citizenship and its indirect relationships through empathy and mental well-being, while also providing initial psychometric evidence for the Turkish form of the scale.

## 2 Theoretical framework

### 2.1 Internal political efficacy

Political efficacy constitutes a core construct for understanding how individuals interpret both their capacity to influence political processes and the responsiveness of political institutions to citizens’ demands. Early applications of the concept originated in studies of voter behavior, which underscored the significance of citizens’ perceived ability to influence political processes as a prerequisite for democratic participation (Campbell et al., 1954). The distinction between personal political competence and perceptions of institutional responsiveness was subsequently articulated more clearly in the classical literature (Lane, 1959), and Balch (1974) provided empirical support for distinguishing internal and external dimensions of political efficacy. Craig and Maggiotto (1982) emphasized the need to distinguish between internal efficacy, which refers to individuals’ perceptions of their own political capacity, and external efficacy, which refers to perceptions of the political system’s responsiveness to citizens’ demands. Craig et al. (1990), in the context of the NES pilot study, examined items on political efficacy and political trust, demonstrating that internal efficacy can be measured robustly and that external efficacy can be distinguished from political trust. Niemi et al. (1991) subsequently evaluated four new internal-political-efficacy items included in the 1988 National Election Study, further sharpening the measurement of this dimension. Within this distinction, internal political efficacy refers to the subjective perception of competence in understanding political issues, acquiring knowledge about political processes, and participating effectively in political life (Niemi et al., 1991). Conceptually, this construct differs from external political efficacy, which focuses on the responsiveness of political systems and institutions to citizen demands, whereas internal political efficacy reflects individuals’ belief in their own political capacity. This distinction is important for the study, as global citizenship requires not only awareness of global issues but also the ability to relate these issues to political and public processes.

Internal political efficacy can be associated, from a socio-cognitive perspective, with the idea of individual agency. In Bandura (1989) social-cognitive theory, human agency emphasizes that individuals are not merely passive recipients of environmental conditions. Instead, they are subjects capable of regulating their own thoughts, motivations, and actions, and of believing that they can influence the outcomes of their behavior. Applied to the political domain, this perspective suggests that individuals’ belief in their ability to understand public issues and assume a meaningful role in relation to them may support citizenship orientations. In the political participation literature, Finkel (1985) demonstrated that participation experiences and perceptions of political efficacy may be reciprocally related. Reichert (2016) showed that political knowledge can be transformed into political participation through internal political efficacy. Prats and Meunier (2021) also reported positive associations between internal political efficacy and diverse forms of political engagement. The collective action literature also indicates that perceived efficacy and social identity processes can support individuals’ tendency to act in response to shared problems (van Zomeren et al., 2008). Within this framework, internal political efficacy is considered one of the key psychological antecedents that explains, in the context of global citizenship, not only individuals’ awareness of global issues but also their ability to see themselves as effective and responsible actors in relation to these issues.

### 2.2 Empathy

Empathy is regarded as a fundamental socio-emotional capacity that enables individuals to develop sensitivity to others’ emotions, experiences, and perspectives. The empathy literature demonstrates that the concept comprises multiple components, such as cognitive perspective-taking and emotional responsiveness (Davis, 1983). The positive relationship between empathy and prosocial behaviors has long been discussed as an important finding. Although this relationship may vary depending on the measurement method employed, it generally exhibits a low-to-moderate positive pattern (Eisenberg and Miller, 1987). Within this framework, empathy can be considered an important psychological resource not only in interpersonal relations but also in broader public and citizenship orientations.

In the original development study of the Toronto Empathy Questionnaire (TEQ) used in this research, empathy was conceptualized particularly in terms of the tendency to provide emotional responses to others’ experiences (Spreng et al., 2009). Accordingly, in the study, empathy was addressed primarily in the context of emotional sensitivity and the tendency to respond to others’ experiences.

Empathy is particularly significant in the context of global citizenship, as global issues often concern the living conditions, rights deprivations, and needs of people beyond the individual’s immediate sphere of experience. Reysen and Katzarska-Miller (2013) demonstrated that global citizenship identity is associated with prosocial orientations such as intergroup empathy, valuing diversity, social justice, environmental sustainability, helping, and responsibility for action. Carmona et al. (2024) further showed that cognitive empathy is related to engagement with global issues and that global citizenship identity plays a partial mediating role in this relationship. This study extends the aforementioned literature by focusing on the emotional dimension of empathy and proposes that empathy may constitute a meaningful indirect pathway between internal political efficacy and global citizenship.

### 2.3 Mental well-being

Mental well-being is not merely the absence of psychological problems. Instead, it is a broad construct encompassing positive psychological resources such as positive affect, functioning, meaning, the ability to form relationships, clarity of thought, and coping with daily life. Keyes (2002) conceptualizes mental health in terms of positive emotions and positive functioning, whereas Ryff (1989) defines psychological well-being through dimensions such as self-acceptance, positive relations, autonomy, environmental mastery, purpose in life, and personal growth. Ryan and Deci (2001) evaluate the well-being literature within the framework of hedonic and eudaimonic approaches. Tennant et al. (2007) developed the Warwick-Edinburgh Mental Well-Being Scale to assess both hedonic and eudaimonic aspects of positive mental health.

The inclusion of mental well-being as a mediating variable in the present model rests on two rationales. First, internal political efficacy may support individuals’ sense of control, competence, and meaning. Those who perceive themselves as more politically competent may feel less helpless and more agentic in the face of public issues. Second, mental well-being may facilitate individuals’ sustainable engagement with global and public issues. Addressing global problems requires not only knowledge and sensitivity but also emotional resilience, cognitive clarity, and a sense of purpose.

The literature on civic engagement and well-being provides a foundation for considering mental well-being as a supportive psychological resource in public participation processes. Fenn et al. (2021) demonstrated that the relationship between civic engagement and well-being is neither direct nor uniform, and that psychological mechanisms such as service-related self-efficacy and meaning in life may play a role in this relationship. Chan et al. (2021) showed that the relationship among social movement participation, political efficacy, and well-being is longitudinally variable and sensitive to political context. Accordingly, mental well-being is examined as a potential psychological pathway linking internal political efficacy with global citizenship.

### 2.4 Global citizenship

Global citizenship is a multidimensional citizenship orientation associated with individuals’ perception of themselves as part of a broader human community, their development of sensitivity to global issues, and their assumption of responsibility for addressing common problems. United Nations Educational, Scientific and Cultural Organization [UNESCO] (2015) relates global citizenship education to cognitive, socio-emotional, and behavioral domains of learning, whereas Organisation for Economic Co-operation and Development [OECD] (2018) defines global competence as the capacity to examine local, global, and intercultural issues, to understand different perspectives, to engage in intercultural interaction, and to take action for collective well-being. These approaches demonstrate that global citizenship extends beyond the mere possession of knowledge. It encompasses values, sensitivity, competence, and orientations toward action.

Morais and Ogden (2011) conceptualized global citizenship through three core dimensions: social responsibility, global competence, and global civic engagement. Social responsibility refers to personal accountability for global injustices, inequalities, and the global consequences of local behaviors; global competence involves openness to understanding different cultural norms and expectations, intercultural communication, and knowledge of global issues; and global civic engagement is associated with the tendency to respond to local, national, and global issues through volunteering, political voice, and public action. Lo et al. (2016) developed a 15-item short form based on this construct, and Gezer and İlhan (2023) provided validity and reliability evidence for the three-dimensional structure of the Turkish short form. Accordingly, in the study, global citizenship is treated as a multidimensional latent variable represented by the subdimensions of social responsibility, global competence, and global civic engagement.

### 2.5 Empathy and mental well-being as parallel psychological pathways

Taken together, the preceding sections suggest that empathy and mental well-being represent conceptually distinct but potentially complementary processes in the relationship between internal political efficacy and global citizenship. Empathy reflects socio-emotional sensitivity to others’ experiences, whereas mental well-being reflects positive psychological functioning and resources. These complementary perspectives provide a theoretical basis for considering empathy and mental well-being as parallel psychological pathways between internal political efficacy and global citizenship. The analytical rationale for specifying the pathways in parallel rather than serially is presented in section “4.1 Research design.”

## 3 Research purpose and hypotheses

Previous research has documented links between internal political efficacy and political participation (Reichert, 2016; Prats and Meunier, 2021), political self-efficacy and global citizenship outcomes (Liu et al., 2023), empathy and engagement with global issues (Carmona et al., 2024), and civic engagement, political efficacy, and well-being (Chan et al., 2021; Fenn et al., 2021). However, these relationships have largely been examined separately, and limited evidence is available on how internal political efficacy, empathy, mental well-being, and global citizenship operate together within a single structural model. In particular, the roles of empathy and mental well-being as distinct but complementary parallel psychological pathways between internal political efficacy and global citizenship remain insufficiently examined. In addition to this substantive gap, a validated Turkish version of the Internal Political Efficacy Scale developed by Niemi et al. (1991) has not been available, limiting its use in Turkish samples.

The study addresses these two gaps through two consecutive stages. First, the four-item Internal Political Efficacy Scale was adapted into Turkish, and its unidimensional structure and reliability were examined using a separate sample. Second, the direct relationship between internal political efficacy and global citizenship and the parallel indirect relationships through empathy and mental well-being were tested using structural equation modeling in a second sample. Due to the cross-sectional design of the study, the hypotheses express direct and indirect relationships consistent with the theoretical model rather than causal effects (Maxwell et al., 2011). In line with these aims, the following hypotheses were tested:

H1: There is a positive relationship between internal political efficacy and global citizenship.

H2: There is a positive relationship between internal political efficacy and empathy.

H3: There is a positive relationship between internal political efficacy and mental well-being.

H4: There is a positive relationship between empathy and global citizenship.

H5: There is a positive relationship between mental well-being and global citizenship.

H6: There is an indirect pathway between internal political efficacy and global citizenship through empathy.

H7: There is an indirect pathway between internal political efficacy and global citizenship through mental well-being.

## 4 Method

### 4.1 Research design

This study employed a quantitative, cross-sectional research design that included both scale adaptation and relational model testing stages. In the first stage, the unidimensional structure and reliability of the Turkish form of the Internal Political Efficacy Scale were examined. In the second stage, the direct relationship between internal political efficacy and global citizenship, as well as the indirect pathways through empathy and mental well-being, were tested using structural equation modeling within the framework of a parallel mediation model. Due to the cross-sectional design, the findings were interpreted not as causal evidence but as structural relationships and potential mediation patterns consistent with the theoretical model (Maxwell et al., 2011).

The parallel specification was selected because the theoretical evidence does not establish a defensible temporal ordering between empathy and mental well-being, while cross-sectional mediation models cannot determine temporal precedence or causal ordering (Maxwell et al., 2011). Multiple-mediation models allow the specific indirect pathways through more than one mediator to be estimated simultaneously without imposing a serial sequence (Preacher and Hayes, 2008).

### 4.2 Participants and data collection

The research used two independent samples comprising 681 participants. The psychometric analyses of the Turkish form of the Internal Political Efficacy Scale were conducted with 311 participants, of whom 132 were women, 157 were men, and 22 preferred not to disclose their gender. The structural model was tested with data from 370 participants, of whom 225 were women, 140 were men, and 5 preferred not to disclose their gender.

Both samples comprised voluntary adult participants aged 18 years or older residing in Türkiye. Data were collected separately for the two analytical stages. For the psychometric analyses (*n* = 311), data were collected through both an online questionnaire administered via Google Forms and face-to-face manual administration. For the structural model analyses (*n* = 370), data were collected exclusively online via Google Forms.

The research process was conducted in accordance with the principles of scientific research and publication ethics. Ethical approval for the study was obtained from the Scientific Research and Publication Ethics Committee of Artvin Çoruh University. At its meeting on December 10, 2025, the committee unanimously decided that the study complied with scientific research and publication ethics. The official date and number of the ethics approval document are 12.12.2025-204948. Prior to data collection, participants were informed about the purpose of the study, the voluntary basis of participation, confidentiality principles, and their right to withdraw from the study at any stage. Informed consent was obtained from all participants.

### 4.3 Measures

#### 4.3.1 Internal political efficacy scale

The Internal Political Efficacy Scale used in this study is a four-item instrument developed by Niemi et al. (1991). The scale assesses individuals’ subjective perception of their competence in understanding political issues and participating effectively in political processes. The original study reported that the four items measured internal political efficacy as a unidimensional construct and distinguished it from external political efficacy and political trust. Psychometric analyses of the Turkish form of the scale were conducted using data obtained from 311 participants. The unidimensional structure of the Turkish form was tested through confirmatory factor analysis, and its internal consistency was evaluated using Cronbach’s alpha, McDonald’s omega, and Guttman’s lambda-6 coefficients.

In preparing the Turkish form of the Internal Political Efficacy Scale, an adaptation process was followed that included translation, back-translation, expert review, and pilot testing. During this process, the conceptual consistency, linguistic clarity, and cultural appropriateness of the items with the original form were evaluated. The final Turkish version was developed based on expert opinions and pilot study results. Recommendations for ensuring the conceptual, semantic, and content equivalence of self-report scales in the target language and culture were taken into account during the translation and cross-cultural adaptation process (Beaton et al., 2000; Sousa and Rojjanasrirat, 2011). In the psychometric evaluation of the Turkish form, principles recommended for scale development and adaptation studies in terms of factor structure, reliability, and validity analyses were followed (Boateng et al., 2018; Worthington and Whittaker, 2006).

#### 4.3.2 Toronto Empathy Questionnaire

The Toronto Empathy Questionnaire is a unidimensional, five-point Likert-type self-report scale developed by Spreng et al. (2009) to assess individuals’ empathic tendencies. Psychometric evidence for the Turkish form of the scale was provided by Totan et al. (2012). In the study, the unidimensional 13-item Toronto Empathy Questionnaire was used, with negatively worded items reverse-coded prior to analysis. An example of such an item is the statement, “Other people’s misfortunes do not affect me very much.”

#### 4.3.3 Warwick-Edinburgh Mental Well-Being Scale

The Warwick-Edinburgh Mental Well-Being Scale is a unidimensional, 14-item, five-point Likert-type self-report instrument developed by Tennant et al. (2007) to assess aspects of mental well-being including positive affect, psychological functioning, and interpersonal relationships. The scale encompasses both hedonic and eudaimonic dimensions of positive mental health and consists solely of positively worded items. The validity and reliability study of the Turkish form was conducted by Keldal (2015), who reported that the Turkish version has a unidimensional structure and is used as a valid and reliable measure of mental well-being. The scale contains no reverse-coded items, and higher scores indicate higher levels of mental well-being.

#### 4.3.4 Global citizenship scale-short form

The Global Citizenship Scale – Short Form is a 15-item, five-point Likert-type instrument used to assess individuals’ global citizenship orientations. The theoretical foundation of the scale is based on the dimensions of social responsibility, global competence, and global civic engagement, as articulated in the Global Citizenship Scale developed by Morais and Ogden (2011). The short form was developed by Lo et al. (2016), and the Turkish adaptation was conducted by Gezer and İlhan (2023). The Turkish adaptation study reported that the scale’s three-dimensional structure was confirmed and that it produced valid and reliable measurements.

### 4.4 Data analysis

Data analysis in the study was conducted in two main stages. In the first stage, psychometric evidence regarding the Turkish form of the Internal Political Efficacy Scale was examined. In the second stage, the direct relationship between internal political efficacy and global citizenship, as well as the parallel mediating roles of empathy and mental well-being in this relationship, were tested through structural equation modeling. Analyses were carried out in the R 4.4.3 environment using version 0.6–21 of the lavaan package (R Core Team, 2025; Rosseel, 2012).

In the first stage, the measurement model of the Turkish form of the Internal Political Efficacy Scale was tested using confirmatory factor analysis with data obtained from 311 participants. Model fit was evaluated with χ^2^/df, CFI, TLI, IFI, GFI, RMSEA, and SRMR indices. Reliability was assessed using Cronbach’s alpha, McDonald’s omega, and Guttman’s lambda-6 (λ_6_) coefficients. Item discrimination was further examined through corrected item-total correlations and Cronbach’s alpha coefficients when items were deleted. Prior to analysis, the dataset was checked for missing values, and none were found. In interpreting fit indices, theoretical appropriateness and multiple fit indicators were considered together rather than relying mechanically on a single cutoff value (Hu and Bentler, 1999; Kline, 2016).

In the second stage, structural equation modeling analyses were conducted using the second dataset of 370 participants. First, the measurement model was tested, followed by examination of the direct relationship between internal political efficacy and global citizenship, and the parallel mediating roles of empathy and mental well-being. In constructing indicators for the measurement model, the number of items and theoretical structures of the scales were taken into account. Since the Internal Political Efficacy Scale consists of a short four-item form, its items were used directly as indicators of the latent variable. For the Toronto Empathy Questionnaire and the Warwick-Edinburgh Mental Well-Being Scale, three content-balanced item parcels were created for each, considering their unidimensional structures and item counts. The Global Citizenship Scale was parceled based on its theoretical subdimension structure. Scores for social responsibility, global competence, and global civic engagement were used as three indicators of the latent global citizenship variable. Item parceling involves combining two or more items measuring the same latent construct into total or mean scores to be used as indicators (Matsunaga, 2008). Parceling can be preferred in scales with theoretically and psychometrically supported unidimensionality to reduce model complexity and obtain more stable parameter estimates, but it must be used cautiously as it may mask model errors in multidimensional or structurally ambiguous item sets (Bandalos, 2002; Little et al., 2013).

Model acceptability was assessed through the standardized factor loadings of the observed indicators on their latent constructs and by examining overall model fit indices. After the measurement model demonstrated acceptable fit, the structural model was tested. In the structural model, the direct path between internal political efficacy and global citizenship, as well as the indirect paths through empathy and mental well-being, were examined simultaneously. Gender was included in the model as a control variable. Model fit was evaluated using χ^2^/df, NFI, GFI, IFI, CFI, TLI, RMSEA, and SRMR indices. The full mediation model was compared with the partial mediation model using chi-square difference tests, fit indices, and information criteria including AIC, BIC, and ECVI. Since the sampling distribution of indirect effects in multiple mediation models may not rely on normality assumptions, the significance of indirect relationships was tested using 5,000 bootstrap resamples and confidence intervals (Preacher and Hayes, 2008). Confidence intervals that did not include zero were interpreted as evidence of statistically significant indirect effects. To evaluate the adequacy of the sample size for testing model fit, a post-hoc power analysis was conducted using the semPower package, which enables power analysis in structural equation modeling (Moshagen and Bader, 2024). The power analysis was performed under the conditions RMSEA = 0.05, α = 0.05, df = 77, and *N* = 370.

## 5 Results

### 5.1 Findings on the Turkish adaptation of the internal political efficacy scale

The four-item structure of the Internal Political Efficacy Scale was examined through confirmatory factor analysis. The model demonstrated good fit with the data: χ^2^(2, *N* = 311) = 1.942, *p* = 0.379, χ^2^/df = 0.971, NFI = 1.000, CFI = 1.000, TLI = 1.000, IFI = 1.000, GFI = 1.000, RMSEA = 0.000, and SRMR = 0.011. Standardized factor loadings were 0.656 for item IPE1, 0.902 for item IPE2, 0.740 for item IPE3, and 0.705 for item IPE4. Skewness values ranged from –0.104 to 0.195, and kurtosis values ranged from –1.194 to –0.829. The reliability coefficients of the scale were Cronbach’s alpha = 0.806, McDonald’s omega = 0.841, and Guttman’s lambda-6 = 0.772. In addition, the composite reliability coefficient was CR = 0.840, and the average variance extracted was AVE = 0.572. Regarding sample adequacy, the KMO coefficient was 0.780 based on Pearson correlations and 0.790 based on polychoric correlations. Bartlett’s test of sphericity yielded a significant result, χ^2^(6) = 496.894, *p* <0.001. Corrected item-total correlations and Cronbach’s alpha coefficients if item deleted are presented in Table 1. These findings indicate that the four-item unidimensional structure of the Turkish form of the scale is consistent with the data and demonstrates acceptable reliability.

**TABLE 1:** Corrected item-total correlations of the internal political efficacy scale.

Item	Corrected item-total r	α if item deleted
IPE1	0.550	0.794
IPE2	0.739	0.706
IPE3	0.623	0.757
IPE4	0.592	0.772

Corrected item-total correlations ranged from 0.550 to 0.739. When items were deleted, Cronbach’s alpha coefficients remained between 0.706 and 0.794, and the removal of any item did not increase the overall alpha coefficient (α = 0.806). These results indicate that all four items contribute adequately to the scale and that retaining them is appropriate.

### 5.2 Descriptive statistics, correlations, and reliability analyses

Table 2 presents descriptive statistics, normality indicators, reliability coefficients, and Pearson correlations for the study variables. Skewness values ranged from -0.830 to -0.085, and kurtosis values ranged from -0.791 to 0.443. These values indicate that the scores did not deviate substantially from a univariate normal distribution. A positive and low-level significant relationship was found between internal political efficacy and empathy (*r* = 0.242, *p* < 0.001). Internal political efficacy was positively and moderately associated with mental well-being (*r* = 0.493, *p* < 0.001) and global citizenship (*r* = 0.441, *p* < 0.001). Positive and moderate significant relationships were also identified between empathy and mental well-being (*r* = 0.439, *p* < 0.001), and between empathy and global citizenship (*r* = 0.441, *p* < 0.001). The relationship between mental well-being and global citizenship was likewise positive, moderate, and significant (*r* = 0.482, *p* < 0.001). McDonald’s omega coefficients ranged from 0.796 to 0.955, Cronbach’s alpha coefficients from 0.792 to 0.953, and Guttman’s lambda-6 coefficients from 0.807 to 0.961. These findings indicate that the measurement instruments used in the study demonstrated internal consistency levels ranging from acceptable to high.

**TABLE 2:** Descriptive statistics, normality indicators, reliability coefficients, and correlations of the study variables.

Variable	1	2	3	4
1. Internal political efficacy	–			
2. Empathy	0.242***	–		
3. Mental well-being	0.493***	0.439***	–	
4. Global citizenship	0.441***	0.441***	0.482***	–
Mean	2.878	3.968	3.572	2.375
SD	0.993	0.613	0.900	0.629
Skewness	−0.085	−0.334	−0.830	−0.141
Kurtosis	−0.583	−0.791	0.443	−0.220
McDonald’s ω	0.841	0.796	0.955	0.907
Cronbach α	0.836	0.792	0.953	0.849
Guttman λ6	0.807	0.828	0.961	0.900

*p < 0.05,

**p < 0.01,

***p < 0.001.

### 5.3 Measurement model

Analysis of the measurement components within the structural model revealed that the standardized factor loadings of the internal political efficacy items ranged from 0.627 to 0.817. The factor loadings of the empathy parcels ranged from 0.718 to 0.845, while those of the mental well-being parcels ranged from 0.908 to 0.969. The standardized factor loadings of the global citizenship subdimensions were 0.395 for social responsibility, 0.902 for global competence, and 0.446 for global civic engagement. All factor loadings were statistically significant (*p* < 0.001). These findings indicate that, in this sample, the latent construct of global citizenship was predominantly represented by the global competence dimension, while the social responsibility and global civic engagement indicators also contributed significantly and in the same direction to the construct. The standardized factor loadings of the measurement model are presented in Table 3.

**TABLE 3:** Standardized factor loadings of the measurement model.

Latent variable	Indicator	Standardized loading (λ)
IPE	IPE1	0.627
IPE	IPE2	0.817
IPE	IPE3	0.760
IPE	IPE4	0.809
TEQ	TEQ1	0.800
TEQ	TEQ2	0.718
TEQ	TEQ3	0.845
WEMWBS	WEMWBS1	0.969
WEMWBS	WEMWBS2	0.937
WEMWBS	WEMWBS3	0.908
GCS	SR	0.395
GCS	GC	0.902
GCS	GCE	0.446

IPE indicators are scale items; TEQ and WEMWBS indicators are item parcels; GCS indicators are subdimension scores. *p* < 0.001 for all loadings.

The measurement model demonstrated acceptable fit with the data: χ^2^(59, *N* = 370) = 142.968, *p* < 0.001, χ^2^/df = 2.423, NFI = 0.951, GFI = 0.993, IFI = 0.971, CFI = 0.970, TLI = 0.961, RMSEA = 0.062, 90% CI [0.049, 0.075], and SRMR = 0.042. No negative error variances or standardized factor loadings exceeding 1.00 were observed. The highest standardized factor loading was 0.969, and the lowest standardized error variance was 0.061. These findings demonstrate that no technical Heywood problem was present in the model solution.

### 5.4 Parallel mediation analysis

To examine the parallel mediating roles of empathy and mental well-being in the relationship between internal political efficacy and global citizenship, full and partial mediation models were compared. The full mediation model, in which the direct path from internal political efficacy to global citizenship was constrained, demonstrated acceptable fit: χ^2^(78) = 209.353, χ^2^/df = 2.684, NFI = 0.930, GFI = 0.991, IFI = 0.955, CFI = 0.955, TLI = 0.939, RMSEA = 0.067, and SRMR = 0.049. The partial mediation model, in which the direct path was freely estimated, produced better fit indices: χ^2^(77, *N* = 370) = 175.099, *p* < 0.001, χ^2^/df = 2.274, NFI = 0.942, GFI = 0.992, IFI = 0.966, CFI = 0.966, TLI = 0.954, RMSEA = 0.059, 90% CI [0.047, 0.070], and SRMR = 0.040. The chi-square difference test revealed that the partial mediation model fit significantly better than the full mediation model, Δχ^2^(1) = 34.254, *p* < 0.001. The AIC, BIC, and ECVI values of the partial mediation model (10145.061, 10352.477, and 0.760, respectively) were lower than those of the full mediation model (10177.31, 10380.82, and 0.847). Since the direct path from internal political efficacy to global citizenship was also positive and significant in the partial model (β = 0.353, *p* < 0.001), the gender-controlled partial parallel mediation model was preferred. Table 4 presents the findings summarizing the model comparisons.

**TABLE 4:** Comparison of full and partial mediation models.

Model	χ^2^ (df)	χ^2^/df	CFI/TLI	RMSEA/SRMR
Full mediation	209.353 (78)	2.684	0.955/0.939	0.067/0.049
Partial mediation	175.099 (77)	2.274	0.966/0.954	0.059/0.040

Δχ^2^(1) = 34.254, *p* < 0.001.

To evaluate the adequacy of the sample size, a post-hoc power analysis was conducted. Under the conditions RMSEA = 0.05, α = 0.05, df = 77, and *N* = 370, the statistical power was calculated as 0.996. This finding indicates that the current sample size provided sufficient power to test the gender-controlled structural equation model.

According to the standardized path coefficients, internal political efficacy was positively and significantly related to empathy (β = 0.343, *p* < 0.001). The path between internal political efficacy and mental well-being was also positive and significant (β = 0.564, *p* < 0.001). The path from empathy to global citizenship was β = 0.345 (*p* < 0.001), while the path from mental well-being to global citizenship was β = 0.268 (*p* = 0.001). When the mediators and gender control variables were included in the model, the direct path from internal political efficacy to global citizenship remained significant (β = 0.353, *p* < 0.001). The standardized coefficients of the gender-controlled partial parallel mediation model are presented in Figure 1.

**FIGURE 1:**
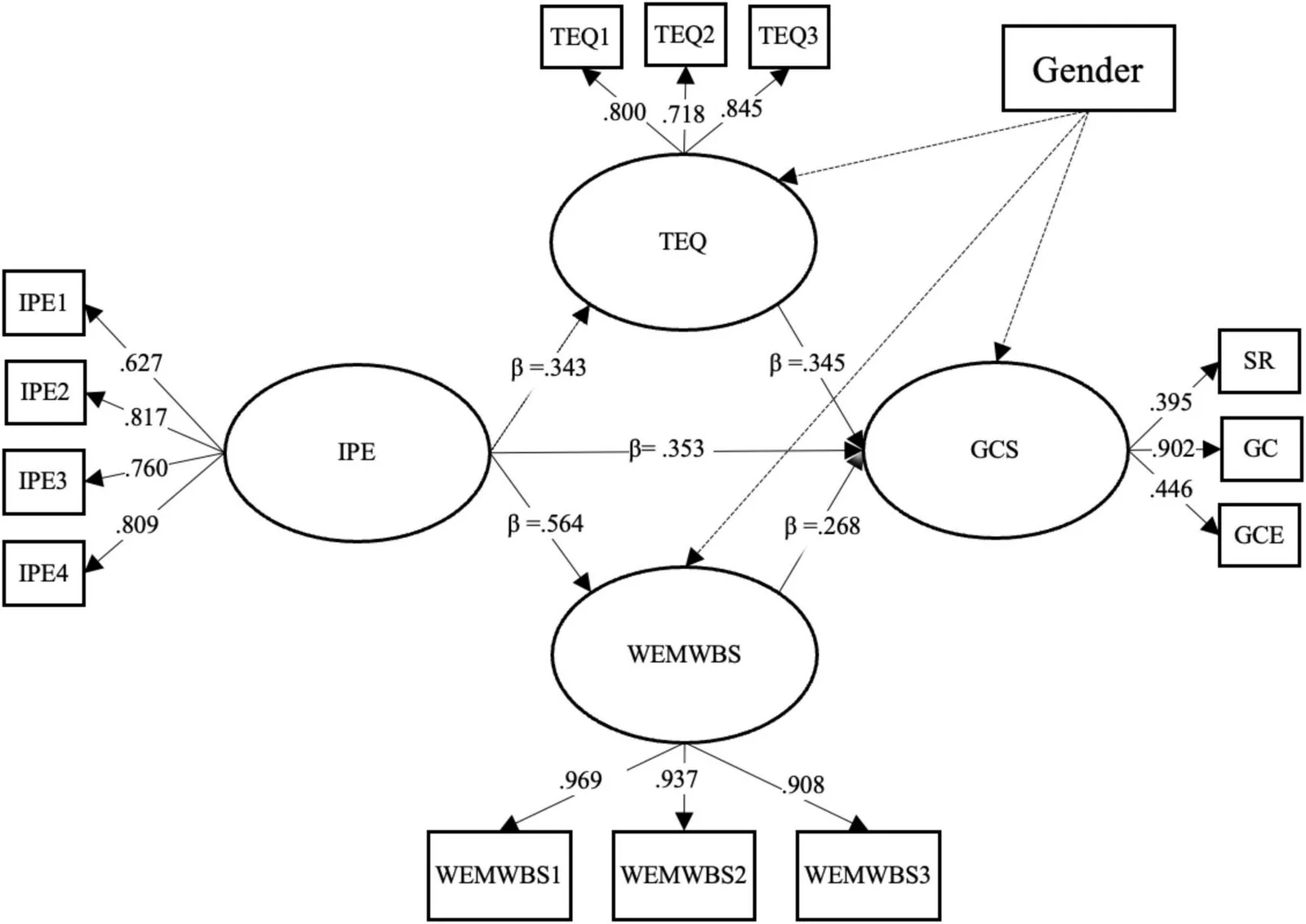
Gender-controlled partial parallel mediation model of internal political efficacy, empathy, mental well-being, and global citizenship. The figure displays standardized structural coefficients and standardized measurement loadings. IPE, internal political efficacy; TEQ, Toronto Empathy Questionnaire; WEMWBS, Warwick-Edinburgh Mental Well-Being Scale; GCS, Global Citizenship Scale; SR, social responsibility; GC, global competence; GCE, global civic engagement. Dashed arrows represent control paths.

When the effect of gender was controlled, internal political efficacy positively predicted empathy (β = 0.343), mental well-being (β = 0.564), and global citizenship (β = 0.353). Empathy (β = 0.345) and mental well-being (β = 0.268) also positively predicted global citizenship.

The model, including gender as a control variable, explained 16.8% of the variance in empathy (R^2^ = 0.168), 31.1% of the variance in mental well-being (R^2^ = 0.311), and 60.6% of the variance in global citizenship (R^2^ = 0.606).

Bootstrap results showed that the indirect path from internal political efficacy to global citizenship through empathy was significant (*B* = 0.039, SE = 0.013, β = 0.118, 95% CI [0.019, 0.071], *p* = 0.003). The indirect path through mental well-being was also significant (*B* = 0.050, SE = 0.016, β = 0.151, 95% CI [0.024, 0.089], *p* = 0.002). The total indirect effect was significant (*B* = 0.089, SE = 0.023, β = 0.269, 95% CI [0.050, 0.144], *p* < 0.001), as was the total effect (*B* = 0.205, SE = 0.039, β = 0.622, 95% CI [0.135, 0.289], *p* < 0.001). The persistence of the direct effect further indicates that empathy and mental well-being serve as partial and parallel mediators in the relationship between internal political efficacy and global citizenship. Bootstrap results for direct, indirect, and total effects are summarized in Table 5.

**TABLE 5:** Bootstrap results for direct, indirect, and total pathways.

Pathway	B (SE)	β	*p*	95% bootstrap CI
IPE → TEQ → GCS	0.039 (0.013)	0.118	0.003	[0.019, 0.071]
IPE → WEMWBS → GCS	0.050 (0.016)	0.151	0.002	[0.024, 0.089]
Total indirect pathway	0.089 (0.023)	0.269	< 0.001	[0.050, 0.144]
Direct pathway: IPE → GCS	0.117 (0.027)	0.353	< 0.001	[0.071, 0.179]
Total pathway	0.205 (0.039)	0.622	< 0.001	[0.135, 0.289]

B, unstandardized coefficient; SE, bootstrap standard error; β, standardized coefficient; CI, confidence interval. Confidence intervals refer to the B coefficients.

## 6 Discussion

First, internal political efficacy was positively related to global citizenship, and the direct path remained positive after empathy, mental well-being, and gender were included in the model (*B* = 0.117, SE = 0.027, β = 0.353, *p* < 0.001, 95% bootstrap CI [0.071, 0.179]). This finding suggests that individuals’ perception of their ability to understand political issues and play a meaningful role in political processes may be connected to broader citizenship orientations. The political efficacy literature has emphasized that internal efficacy reflects a distinct dimension of perceived competence in understanding politics and participating effectively in political life (Craig et al., 1990), that political knowledge can translate into political participation through internal political efficacy (Reichert, 2016), and that participation experiences and efficacy perceptions can mutually reinforce each other, with participation also exerting positive effects on external political efficacy (Finkel, 1985). The present findings extend this body of work to the context of global citizenship, suggesting that perceptions of political competence may also be linked to cross-border responsibility and sensitivity to global issues.

Second, the empathy pathway was supported by the positive paths from internal political efficacy to empathy and from empathy to global citizenship, together with the specific indirect effect through empathy (*B* = 0.039, SE = 0.013, β = 0.118, *p* = 0.003, 95% bootstrap CI [0.019, 0.071]). Empathy may enable individuals to perceive global issues not merely as abstract, knowledge-based concerns but as human problems affecting the lives of diverse communities. Indeed, empathy has generally been found to be positively and moderately related to helping and prosocial behaviors, although these relationships vary depending on the dimension of empathy and the measurement instrument used (Eisenberg and Miller, 1987; Yin and Wang, 2023). The relationship between empathic concern and helping behaviors has been shown to be particularly pronounced in face-to-face and spontaneous informal helping contexts (Einolf, 2008). The association of global citizenship identity with intergroup empathy, social justice, valuing cultural diversity, intergroup helping, and responsibility for action further demonstrates that empathy constitutes one of the core prosocial orientations linked to global citizenship (Reysen and Katzarska-Miller, 2013). Research on identification with humanity has demonstrated that such identification is positively associated with empathic sensitivity, universalism, and justice values, and fosters commitment to human rights as well as attitudes and behaviors aimed at reducing global inequalities (McFarland et al., 2019). Carmona et al. (2024) also found significant relationships among cognitive empathy, global citizenship identity, and engagement with global issues. Thus, empathy may be regarded as a salient socio-emotional mechanism through which perceptions of political competence are linked to orientations toward global responsibility and participation.

Third, the mental well-being pathway was likewise supported by the positive paths from internal political efficacy to mental well-being and from mental well-being to global citizenship, together with the specific indirect effect through mental well-being (*B* = 0.050, SE = 0.016, β = 0.151, *p* = 0.002, 95% bootstrap CI [0.024, 0.089]). This finding aligns with Fenn et al. (2021), who demonstrated that the relationship between civic engagement and well-being can be indirectly shaped by psychological mechanisms such as meaning in life and service-related self-efficacy. Chan et al. (2021) also showed that the relationships among participation in social movements, political efficacy, and well-being vary depending on the form of participation, the political outcomes of the movement, and perceptions of governmental responsiveness to citizens’ demands. Accordingly, mental well-being may be considered a complementary channel representing positive psychological functioning in the relationship between internal political efficacy and global citizenship in the present model.

Fourth, the total indirect effect (*B* = 0.089, SE = 0.023, β = 0.269, *p* < 0.001, 95% bootstrap CI [0.050, 0.144]) was consistent with the joint contribution of the two specific indirect pathways. This pattern is consistent with Reichert (2016) findings that political knowledge can translate into political participation through internal political efficacy. The conceptual analysis by Eidhof and de Ruyter (2022) emphasized that self-efficacy beliefs serve a motivational function in transforming citizenship-related knowledge and skills into action. Studies showing that identification with humanity is related to prosocial orientations such as supporting human rights, intergroup helping, and assuming responsibility in the face of global issues (McFarland et al., 2019), together with findings that meaning in life and service-related self-efficacy play a role in the relationship between civic engagement and well-being (Fenn et al., 2021), support the interpretation that empathy and mental well-being may serve distinct yet complementary functions. The persistence of the direct path after both indirect pathways were included suggests that empathy and mental well-being do not fully account for the association between internal political efficacy and global citizenship and that other cognitive, affective, social, or educational processes may also play a role. Overall, the results reflect partial parallel mediation through empathy and mental well-being. Liu et al. (2023) also demonstrated that political self-efficacy mediates the relationship between social capital and various global citizenship outcomes, although this pattern did not hold in the context of global environmental sustainability. The present model offers a cross-sectional account of associations among internal political efficacy, the two proposed psychological pathways, and global citizenship.

In addition to these substantive findings, the psychometric analyses supported the Turkish form of the Internal Political Efficacy Scale. The fit indices obtained from confirmatory factor analysis indicated good model fit consistent with the criteria suggested by Hu and Bentler (1999) and Schermelleh-Engel et al. (2003), supporting the unidimensional structure of the scale. This result is consistent with the unidimensional structure reported for the original four-item form by Niemi et al. (1991). The internal consistency coefficients were within acceptable thresholds according to the criteria by DeVellis (2017), supporting the reliability of scores obtained from the Turkish form.

## 7 Limitations and future research

The findings of this study should be evaluated within the framework of certain methodological limitations. The reliance on a cross-sectional design requires that the direct and indirect paths identified in the model be interpreted not as causal effects but as statistical associations consistent with the theoretical model. Given that cross-sectional mediation analyses may yield biased estimates of longitudinal direct and indirect effects (Maxwell et al., 2011), future research could extend the present findings by examining the temporal sequencing of variables through longitudinal and, where possible, experimental or quasi-experimental designs. The collection of data from the same participants using self-report instruments within a single measurement process necessitates consideration of the potential for common method variance (Podsakoff et al., 2003). Although latent variable modeling allows random measurement error to be taken into account, it does not by itself eliminate the possibility of common method variance. Future studies could benefit from incorporating behavioral indicators, multiple data sources, and measurements conducted at different time points to provide a more comprehensive test of the model.

Finally, the psychometric evidence obtained for the Turkish form of the Internal Political Efficacy Scale supports its usability in the Turkish context. Nevertheless, in cross-cultural adaptation processes, it is essential to ensure semantic, idiomatic, experiential, and conceptual equivalence between source and target forms (Beaton et al., 2000). Following translation and cultural adaptation, the factor structure, reliability, and validity evidence should be thoroughly evaluated in the target group (Sousa and Rojjanasrirat, 2011). In this regard, examining measurement invariance of the Turkish form across theoretically meaningful groups defined by age, education, occupation, and region with sufficiently large samples is important. Assessing the predictive validity of scale scores for subsequent political and civic participation behaviors using longitudinal data would provide additional evidence of criterion validity (Boateng et al., 2018).

## 8 Conclusion

This study provided psychometric evidence regarding the usability of the four-item Turkish form of the Internal Political Efficacy Scale in terms of internal structure and reliability. It also demonstrated that internal political efficacy is related to global citizenship both directly and indirectly through empathy and mental well-being. The support for the partial parallel mediation model even when gender was controlled indicates that the core pattern of findings was preserved. Taken together, these findings indicate that perceived political competence, empathic sensitivity, and positive psychological functioning are associated with global citizenship within the tested model. The indirect pathways should be interpreted as associations consistent with the proposed model rather than as causal effects. Nevertheless, the findings should be interpreted within the limitations of a cross-sectional and self-report design.

## Data Availability

The raw data supporting the conclusions of this article will be made available by the authors, without undue reservation.
